# Photonics-based treatments: Mechanisms and applications in oral infectious diseases

**DOI:** 10.3389/fmicb.2023.948092

**Published:** 2023-02-10

**Authors:** Shan Huang, Manlin Qi, Yingxue Chen

**Affiliations:** ^1^Department of Stomatology, Zhuhai People's Hospital (Zhuhai Hospital Affiliated with Jinan University), Zhuhai, China; ^2^Department of Oral Implantology, School and Hospital of Stomatology, Jilin University, Changchun, China

**Keywords:** photonics, oral infectious diseases, antibacterial photodynamic therapy, photothermal therapy, photobiomodulation

## Abstract

Infectious diseases remain a serious global challenge threatening human health. Oral infectious diseases, a major neglected global problem, not only affect people’s lifestyles but also have an intimate association with systemic diseases. Antibiotic therapy is a common treatment. However, the emergence of new resistance problems hindered and enhanced the complication of the treatment. Currently, antimicrobial photodynamic therapy (aPDT) has long been the topic of intense interest due to the advantage of being minimally invasive, low toxicity, and high selectivity. aPDT is also becoming increasingly popular and applied in treating oral diseases such as tooth caries, pulpitis, periodontal diseases, peri-implantitis, and oral candidiasis. Photothermal therapy (PTT), another phototherapy, also plays an important role in resisting resistant bacterial and biofilm infections. In this mini-review, we summarize the latest advances in photonics-based treatments of oral infectious diseases. The whole review is divided into three main parts. The first part focuses on photonics-based antibacterial strategies and mechanisms. The second part presents applications for photonics-based treatments of oral infectious diseases. The last part discusses present problems in current materials and future perspectives.

## Introduction

Oral infectious diseases are often ignored, and they threaten human oral health and systemic health. Some oral infectious diseases such as periodontitis become a major public health problem because of their high prevalence, dental and maxillofacial deformation and functional impairment, high dental care cost, and negative impact on general health (Papapanou et al., 2018). These diseases are usually caused by plaque or dysbiosis. Because most oral infectious diseases are deep tissue infections and the complexity of anatomical structures, the treatment effect is often unsatisfactory.

Biophotonics is defined as the science of imaging, detecting, and manipulating biological materials by generating and using light or photons. It is used successfully in medicine and dentistry to help diagnose and treat various diseases (Besegato et al., 2022). Recently, antibacterial photodynamic therapy (aPDT) and photothermal therapy (PTT) and their derived multimodal synergistic treatments have received extensive attention. Due to its strong bactericidal effect and no bacterial resistance, it is also gradually used in the dental field. The development of some photonics-based materials will be beneficial for the clinical treatment of oral infectious diseases. This mini-review will provide a succinct summary of the mechanism and strategies of photonics-based treatment and applications in oral infectious diseases.

## Photonics-based treatment strategies and antibacterial mechanism

### aPDT-based antibacterial strategies and mechanism

Antibacterial photodynamic therapy is a promising strategy to eradicate pathogenic microbes or biofilm and has the advantages of simple operation, wide application, and no drug resistance. aPDT involves three components: light, a photosensitizer (PS), and oxygen. The light source that excites the corresponding PS, usually from visible light to near-infrared spectrum, in which visible light greater than 430 nm is irradiated outside the tooth, can be used for the extinction of *Enterococcus faecalis* in root canal treatment (Cieplik et al., 2016). However, the excitation light is usually in a fixed wavelength band with limited penetration, which limits its application in deep tissue (Lin et al., 2021). To solve this problem, near-infrared-II light with less energy decay and increased tissue depth is recommended (Zhang G. et al., 2021). Electric-driven aPDT using *in situ* chemiluminescence instead of an external light source to activate PSs is another strategy (Liu et al., 2018). Another limitation of aPDT application is due to the hydrophobicity and aggregation of some PSs. Therefore, a series of hydrophilic PSs were developed to improve the above problems (Sun et al., 2019; Gao et al., 2021). Generally, aPDT has broad-spectral antibacterial activity instead of non-selective one, which may kill off beneficial bacteria. Accordingly, targetable aPDT (Im et al., 2021) or promotion of type I photoreactions (Li et al., 2018) were conducted to be a selective antibacterial strategy. The excellent photoantimicrobials should have the following characteristics: positive charge, hydrophilicity, low molecular weight, High ^1^O_2_ quantum yield, photostability, and appropriate therapeutic window (Maisch, 2020). Nowadays, a growing number of researchers pay more attention to aPDT-based synergistic therapies, which include aPDT and antibiotic therapy (Jiang et al., 2020), enzyme-aPDT (Li et al., 2021b; Hu et al., 2022), photodynamic ion therapy (Li J. et al., 2021), aPDT and low-level laser therapy (LLLT) (He et al., 2021), aPDT and Chemotherapy (Zhang et al., 2015; Hamblin, 2016; Chen H. et al., 2019); aPDT and Chemodynamic Therapy (CDT) (Ma et al., 2021), and aPDT gas therapy (Sun J. et al., 2021; Zhu et al., 2021; Zhou et al., 2022). However, remarkably, synergistic aPDT and antibiotic therapy may be a controversial strategy, since low reactive oxygen species (ROS) concentrations are beneficial for bacteria and can induce resistance in the antibiotic-mediated killing of bacteria (Van Acker and Coenye, 2017). Synergistic therapy is generally not only to improve sterilization ability but also to propose personalized treatment solutions for specific clinical problems, such as generating more oxygen to improve aPDT effect or promoting tissue healing or immunomodulatory. Most of the synergistic therapies have excellent antibacterial effects, especially aPDT and antibiotic therapy (Teic, 4.9 logs reduction against *Staphylococcus aureus* (*S. aureus*; Jiang et al., 2020) and aPDT and chemotherapy [C60-fullerene with KI, 4.5 logs reduction against *A. baumanii* and 4.1 logs reduction against Methicillin-resistant *S. aureus* (*MRSA*); Zhang et al., 2015]. The details of the above aPDT-based synergistic therapies are summarized in Table 1.

**Table 1:** Therapeutic methods in coordination with aPDT.

Synergistic therapies	Definition	Example	Dose of materials	Light parameters	Bacteria	Killing efficiency	Reference
Antibiotic therapy 01	Treatment with antibiotics	PMB	0.32 μg mL^−1^	Visible light, 50 mW cm^−2^, 40 min	kana^r^ *E. coli*	3.9 orders of magnitude	Jiang et al. (2020)
			32 μg mL^−1^	Visible light, 40 mW cm^−2^, 40 min	*S. aureus*	3.9 orders of magnitude	
		SMT	32 μg mL^−1^	Visible light, 50 mW cm^−2^, 40 min	kana^r^ *E. coli*	3.2 orders of magnitude	
		Norf	5 ng mL^−1^	Visible light, 50 mW cm^−2^, 40 min	kana^r^ *E. coli*	3.3 orders of magnitude	
		Teic	0.32 μg mL^−1^	Visible light, 40 mW cm^−2^, 40 min	*S. aureus*	4.9 orders of magnitude	
Nanozyme 02	Nanomaterials with enzyme mimicking activities	Co^II^TBPP(bpy)	1 mg mL^−1^	660 nm laser, 1 W cm^−2^, 10 min	*E. coli*	95%	Hu et al. (2022)
					*P. aeruginosa*		
					*B. amyloliquefaciens*		
					*S. aureus*		
Ion therapy 03	Treatment of disruption of bacterial metabolism by metal ions	Cu^2+^, Fe^3+^	Cu^2+^: 2.25%, Fe^3+^: 2.91%	660 nm laser, 0.5 W cm^−2^ for 10 min	*P. gingivalis*	99.87 ± 0.09%,	Li J. et al. (2021)
					*F. nucleatum*	99.57 ± 0.21%,	
					*S. aureus*	99.03 ± 0.24%	
LLLT or PBM 04	Using irradiation with light of low power intensity so that the effects are a response to the light and not due to heat.	Ti/GelMAc/MPDA@Ce6	2 mg mL^−1^	660 nm laser, 1 W cm^−2,^ 10 min (aPDT) 100 mW cm^−2^,10 min (PBM)	*E. coli*	88.55%	He et al. (2021)
					*S. aureus*	85.60%	
Chemotherapy 05	Treatment with chemotherapy drugs or inorganic salts	ZPMAVP	400 μg mL^−1^	660 nm laser, 100 mW cm^−2^, 5 min	*S. aureus*	almost 50%	Chen et al. (2019)
					*E. coli*	almost 100%	
					*MRSA*	almost 100%	
		C60-fullerene with Iodide	C60-fullerene (20 μM) with KI (10 mM)	UVA light, 100 mW cm^−2^, 20 min	*A. baumanii*	4.5 logs	Zhang et al. (2015); Hamblin (2016)
					*MRSA*	4.1 logs	
					*C. albicans*	over 1 log	
CDT 06	Using the Fenton reaction or Fenton-like reaction to generate. OH	CaO_2_/GQDs@ZIF-67	128 μg mL^−1^	LED, 5 W, 40 cm above bacteria	*E. coli*	99.91%	Ma et al. (2021)
					*S. aureus*	99.99%	
Gas therapy 07	Using gaseous signal molecules	UCNP@PCN@LA-PVDF	1 mg mL^−1^	808 nm light, 1 W cm^−2^, 5 min	*P. aeruginosa*	99.64%	Sun J. et al. (2021)
					*S. aureus*	99.63%	

*A. baumanii*, *Acinetobacter baumannii*; *B. amyloliquefaciens*, *Bacillus amyloliquefaciens*; *C. albicans*, *Candida albicans*; CDT, Chemodynamic therapy; Ce6, Chlorin e6; Co^II^TBPP(bpy), complexing porphyrin, Co^2+^, and 4,4′-bipyridine; *E. coli*, *Escherichia coli*; *F. nucleatum*, *Fusobacterium nucleatum*; kana^r^ *E. coli*, *Escherichia coli* with kanamycin resistance; GelMAc, methacrylated gelatin; GQDs, graphene quantum dots; KI: potassium iodide; LED, light-emitting diode; LLLT, Low-level laser therapy; LA, L-arginine; MPDA, mesoporous polydopamine nanoparticles; MRSA, Methicillin-resistant *S. aureus*; Norf, norfloxacn; *P. aeruginosa*, *Pseudomonas aeruginosa*; PBM, photobiomodulation; PCN, porphyrinic MOFs; *P. gingivalis*, *Porphyromonas gingivalis*; PMB, polymyxin B; PVDF, polyvinylidene fluoride; *S. aureus*, *Staphylococcus aureus*; SMT, sulfamethizole; Teic, teicoplanin; UCNPs, upconversion nanoparticles; UVA, ultraviolet A; ZIF, zeolitic imidazolate framework; ZPMAVP, ZIF-8-PAA-MB@AgNPs@Van-PEG

**Figure 1:**
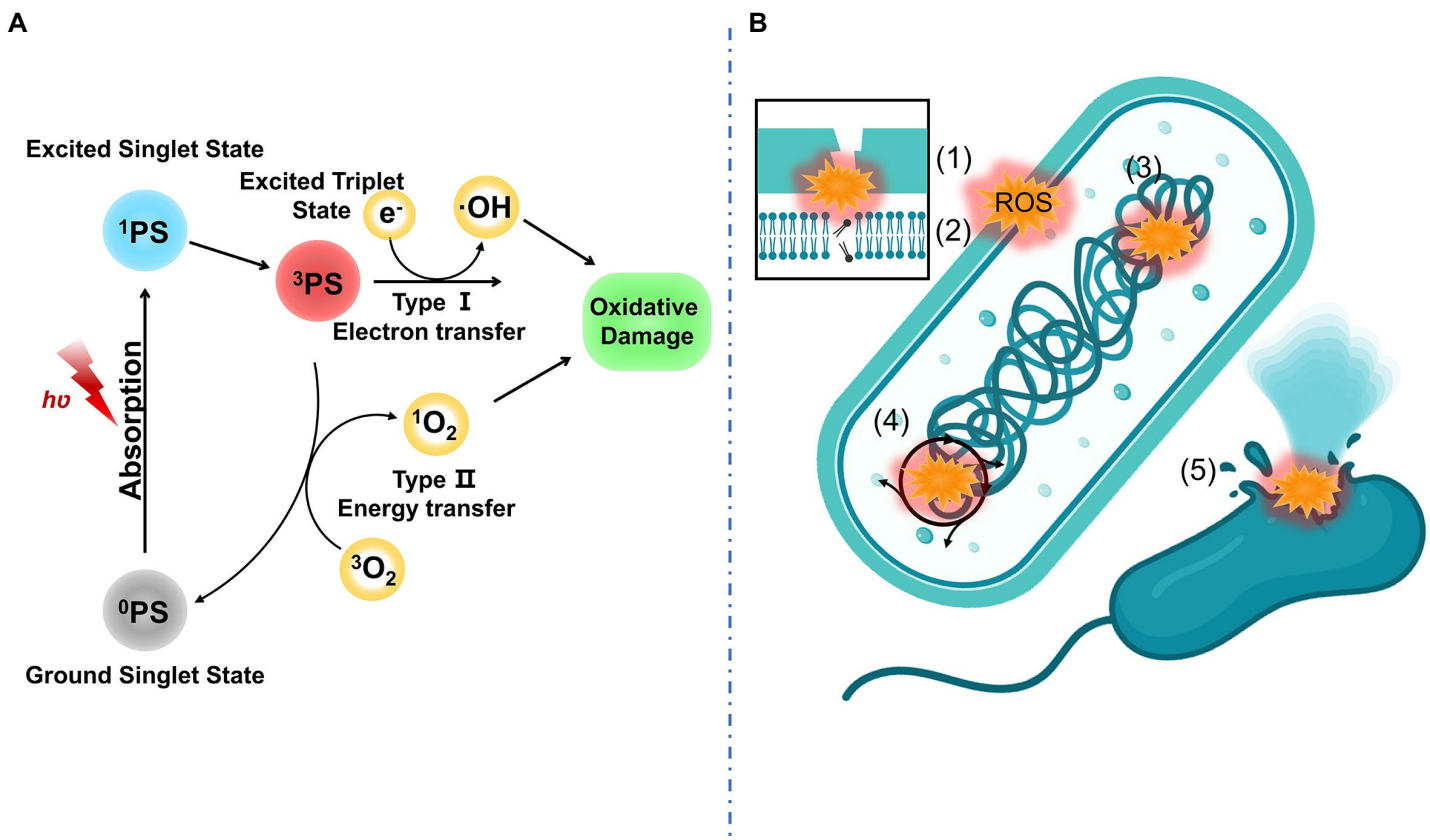
**(A)** PDT mechanism including type I mechanism (electron transfer) and type II mechanism (energy transfer); **(B)** Antibacterial mechanism of aPDT including (1) altered outer membrane permeability, (2) oxidation of lipids, (3) protein or DNA damage, (4) interfere with bacterial metabolism, and (5) irreversible bacterial destruction.

Antibacterial properties of aPDT-based materials are derived from ROS. ROS including superoxide anion (O_2_^.−^), hydrogen peroxide (H_2_O_2_), and hydroxyl radical (OH˙) generated *via* the type I mechanism (electron transfer) and singlet oxygen (^1^O_2_) generated *via* the type II mechanism (energy transfer). The possible antibacterial mechanisms of aPDT may include the following (Liu W. et al., 2020; Fan et al., 2021; Huang et al., 2022): (1) altered outer membrane permeability, (2) oxidation of Lipids, (3) protein or DNA damage, (4) interfere with bacterial metabolism, and (5) irreversible bacterial destruction. These antibacterial mechanisms of aPDT are displayed in Figure 1.

### PTT-based antibacterial strategies and mechanism

PTT is a non-invasive therapy to combat drug-resistant bacteria and plaque biofilm and has the advantages of minimal systemic toxicity, broad-spectrum antibacterial activity, and no drug resistance. When PTT is applied alone, high-power laser excitation and high-dose photothermal agent (PTA) are often required, which may cause tissue damage. Notably, a localized thermal management strategy based on the thermal-disrupting interface-induced mitigation (TRIM) for accurate topical antibacterial therapy was developed (Hu et al., 2020). TRIM film contains a critical dimension for surface features, which leads to species-specific spatial confinement. Bacteria are attached to the microvalleys while host cells can only attach to the microtidges. After infrared irradiation, the phase change material poly(N-isopropylacrylamide) (pNIPAM) polymerized and made the PTA gold nanostars gather near the bacteria without host cell damage. pNIPAM is photothermal-responsive and could transfer from the hydrophilic phase to the hydrophobic phase when the temperature is up to a lower critical solution temperature. Taking advantage of this property, such material could trap bacteria (Yang et al., 2018, 2019). Notably, while PTA exerts its bactericidal properties, the local temperature should be controlled to avoid the aggravation of local tissue inflammation or secondary damage. Therefore, many researchers have proposed the strategy of mild temperature PTT. Usually, a mild temperature is no more than 45°C, which can act as an antibacterial or promote drug release (Ye et al., 2020; Zhang G. et al., 2021). In addition, there are various antibacterial strategies for PTT-based synergistic therapy including synergistic PTT and antibiotic therapy (Huang et al., 2020; Wang et al., 2020), enzyme-PTT (Wang et al., 2021; Xie et al., 2021), photothermal ion therapy (Li et al., 2019), synergistic PTT and CDT (Liu Z. et al., 2021), sonodynamic therapy (SDT) and PTT (Bi et al., 2022), and PTT gas therapy (Liu Y. N. et al., 2020).

**Figure 2:**
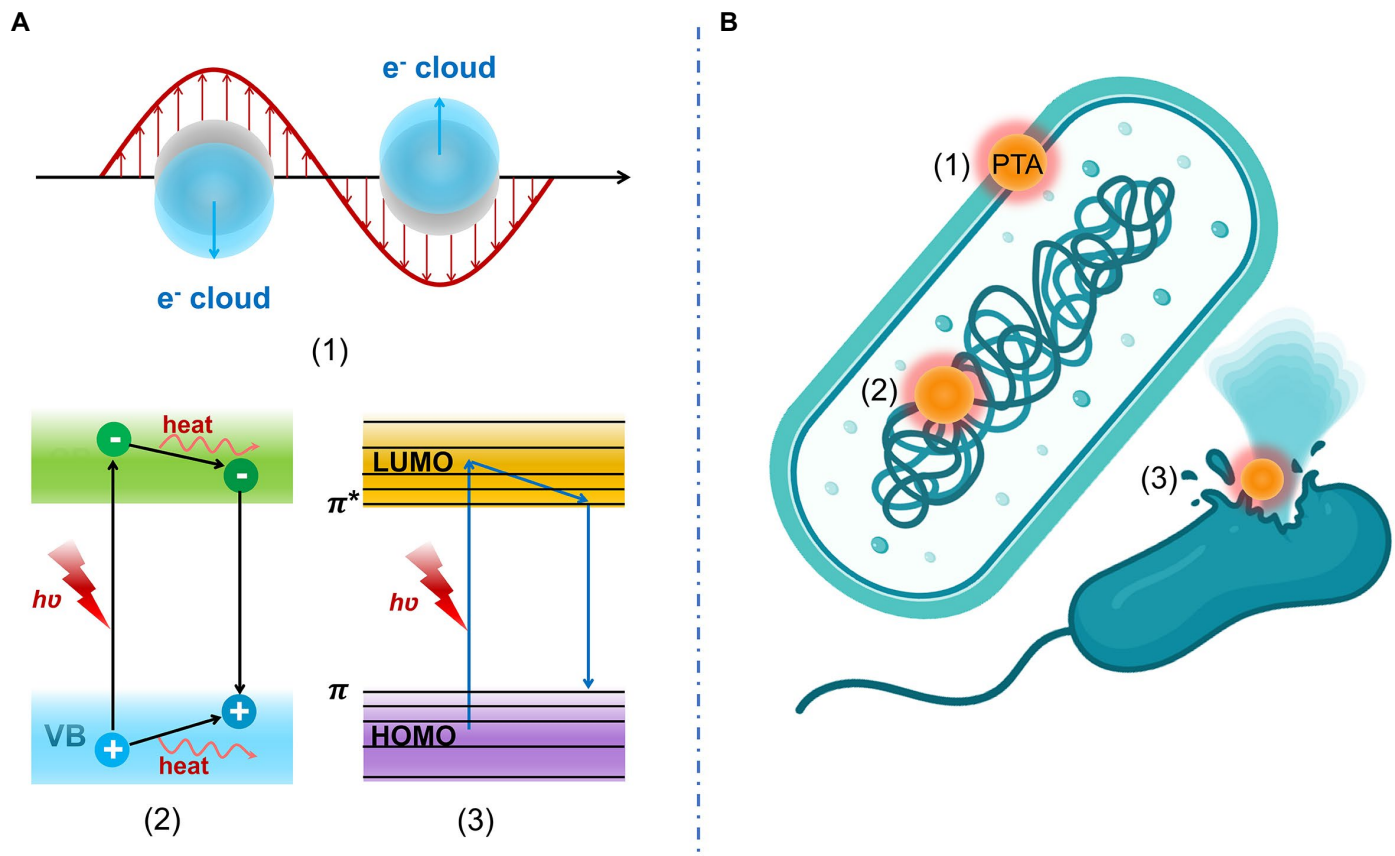
**(A)** Photothermal conversion mechanism including (1) plasmonic heating, (2) electron–hole generation and relaxation, and (3) thermal vibration of molecules. **(B)** Antibacterial mechanisms of PTT include (1) increased membrane permeability, (2) bacterial protein denaturation, and (3) irreversible bacterial destruction.

Generally, PTAs transform light energy into heat energy in three ways including plasmonic heating, electron–hole generation and relaxation, and thermal vibration of molecules (Chen C. J. et al., 2019). Antibacterial mechanisms of PTT include (1) increased membrane permeability, (2) bacterial protein denaturation, and (3) irreversible bacterial destruction. These antibacterial mechanisms of PTT are displayed in Figure 2.

### aPDT- and PTT-derived multimodal synergistic therapies

There were several disadvantages of aPDT or PTT being applied alone. Since ROS has a short half-life and narrow diffusion distance, it can hardly invade into compact bacterial biofilm (Qi et al., 2022). To obtain a good sterilization effect, more dosages of PSs or PTAs and greater light intensity are needed, which may cause tissue damage. To achieve more function and minimal side effects, aPDT- and PTT-derived multimodal synergistic therapies attract attention. Heat can accelerate the release and penetration of drugs, and promote the release of ROS and some chemical reactions. Currently, antibacterial strategies for aPDT/PTT synergistic therapies include synergistic aPDT/PTT (Liu Y. X. et al., 2021; Mo et al., 2022), aPDT/PTT and antibiotic therapy (Zeng et al., 2020), aPDT/PTT and gas therapy (Cai et al., 2021; Qi et al., 2022), aPDT/PTT and ion therapy, and aPDT/PTT/CDT (Zhang J. C. et al., 2021). For aPDT/PTT materials, there are huge differences between different materials in antibacterial activity. The materials with the same composition but different exposed facets could show different antibacterial properties. In Mo’s work, two Cu_7_S_4_ nanosheets with (304) and (224) exposed facets were designed for antibacterial strategy. The antibacterial effects of *Escherichia coli* and *Bacillus subtilis* were 72.2% and 40.7%, respectively, in the former one, while were nearly 100 and 96.8%, respectively, in the latter one. The latter one could produce richer ROS and have more efficient photothermal conversion and stronger adsorption due to different structures (Mo et al., 2022). In the above-mentioned research, it is worth noting that aPDT/PTT/CDT and aPDT/PTT/gas therapy can achieve over 99% sterilization efficiency. It was reported that CuFeO_4_/graphene oxide coatings with aPDT/PTT/CDT functions showed very high antibacterial effciency of 99.94 ± 1.20% against *S. aureus* and 99.57 ± 0.86% against *E. coli*, respectively (Zhang J. C. et al., 2021). In addition, gas therapy (such as CO and NO) can further enhance sterilization by dissipating biofilm. The aPDT/PTT/NO gas group can lead to an additional reduction of about 1.5 log against *Porphyromonas gingivalis* (*P. gingivalis*) biofilm and over 2 log against *Fusobacterium nucleatum* (*F. nucleatum*) biofilm, compared with the aPDT/PTT group (Qi et al., 2022). Interestingly, Cai et al. (2021) revealed similar results that all tested bacteria including *E. coli*, *S. aureus,* and *MRSA* could be additionally reduced over 2 log in the aPDT/PTT/CO gas group than aPDT/PTT group.

### Low-level laser therapy or photobiomodulation and mechanism

Low-Level Laser Therapy or PBM is a term used to describe a medical procedure that uses low-power lasers (0.5 W), sometimes known as “cold lasers,” so that the effects are a result of the light rather than heat. When mitochondria are stimulated by a light source, PBM reacts photochemically with target cells (Dompe et al., 2020). The cytochrome c oxidase enzyme, which is found in the unit IV respiratory chain of the mitochondria, absorbs the application of red light (600–810 nm). As inhibitory nitric oxide is separated from the enzyme by photons, electron transport, mitochondrial membrane potential, and ATP synthesis are all increased. On the other hand, near-infrared light (810–1,064 nm) application stimulates light-sensitive ion channels and raises Ca^2+^ levels; Then, the Ca^2+^ interacts with ROS and cAMP. All these processes promote cell motility, proliferation, and differentiation (de Freitas and Hamblin, 2016; Dompe et al., 2020).

Although most studies concentrate on the intracellular effects of visible and near-infrared light on mitochondrial cytochrome c oxidase, recent research has shown that transforming growth factor (TGF)-b1 and its downstream targets, Human Beta-Defensin-2 (HBD-2), have direct antimicrobial effects and indirectly control inflammation and encourage tissue regeneration in the peri-implant or periodontal tissues. Laser-mediated HBD-2 expression involves the Smad and non-Smad components of the TGF-b1 signaling pathway (Tang et al., 2017).

## Photonics-based treatment of oral infectious diseases

Oral infectious diseases such as caries, pulpitis, periodontal diseases, peri-implantitis, and oral candidiasis are caused by plaque biofilm or dysbacteriosis. Therefore, effective antimicrobials are key to treating these diseases. However, the infection site of oral infectious diseases is deep and the anatomical structure is often complicated, which makes it difficult to completely remove bacterial plaque and the recurrence of infection. aPDT or PTT as a non-invasive treatment plays an important role in oral infectious diseases.

### Dental caries

Antimicrobial photodynamic therapy can effectively inhibit the growth of a variety of microorganisms associated with cariogenic biofilms, including *Streptococcus*, *Streptococcus mutans* (*S. mutans*), *Lactobacillus,* and yeast (Garcia et al., 2021). A nanoplatform composed of assembling toluidine blue O (TBO) and superparamagnetic iron oxide nanoparticles (MagTBO) was designed to achieve enhanced antimicrobial activity (Balhaddad et al., 2021). Under a magnetic field, 2.5% MagTBO microemulsions showed a 6-log reduction of *S. mutans* monospecies biofilm and a 4.5- to 5.5-log reduction of multispecies biofilms. In addition, an amphiphilic and pH-responsive PS (Polyethylene glycol-b-poly(2-(diisopropylamino)ethyl methacrylate)) (MPEG-b-P(PDA) loaded with Ce6 (MPP-Ce6) was developed for inhibiting multispecies cariogenic biofilms (Liu et al., 2022). MPP-Ce6 had over 99% inhibition against *S. mutans*, *Streptococcus sobrinus*, and *Streptococcus sanguinis*. Although numerous laboratory studies have demonstrated the relative effectiveness of aPDT in reducing the number of cariogenic bacteria in biofilms *in vitro*, preclinical (orthotopic models) and clinical studies have shown that bacterial reduction in aPDT is not significant, especially in dentin carious lesions (Reis et al., 2019).

PTT-based researches on dental caries are rare. Recently, removable photothermal antibacterial “warm paste” nanoagents became striking (Xu X. et al., 2022). In brief, polydopamine (PDA), Ag, and glycol chitosan (GCs) were sequentially modified on the surface of Fe_3_O_4_ to form FePAgPG nanoparticles (NPs). In a cariogenic acid environment, due to the pH-responsive effect, FePAgPG NPs targeted cariogenic bacteria. The FePAgPG NPs inhibited over 95% of biofilm formed by *Streptococcus mutants via* the Ag-assisted PTT strategy. Multimodal synergistic treatments could achieve the best antibacterial effect. A pH-responsive nanoplatform had a triple function with synergetic pharmacological therapy and aPDT/PTT for enhanced biofilm eradication and caries prevention (Yu et al., 2022). The combination of ciprofloxacin and IR780 showed an amazing killing rate of 99.8% and considerably high biofilm dispersion of about 70–80%. Moreover, under an acidic oral biofilm microenvironment, the nanoplatform suffered from degradation, anchored bacteria, and released drugs on demand.

### Pulp infection

Incomplete cleaning of infected dental pulp causes failure of endodontic treatment and refractory apical periodontitis. In terms of bacterial control, aPDT has proven to be a positive option as an adjunct to conventional root canal techniques (Lopes et al., 2022). Indocyanine Green (ICG) was regarded as the best PS for endodontic infection compared with TBO and methylene blue (MB). The enterococcal surface protein (*esp*) gene was related to colonization and bacterial resistance in endodontic infections. The expression of *esp* was significantly downregulated to approximately ~5.2-fold by ICG (Chiniforush et al., 2018). Nano-MOF Fe-101 loading ICG (Fe-101-ICG) could downregulate the expression of *esp* to 6.2-fold (Golmohamadpour et al., 2018). Notably, ICG is not only a PS but also a PTA. Some researchers ignored the additional bactericidal effect of the photothermal effect of ICG. Besides, some Zn-based PSs were developed to combat *Enterococcus faecalis* (*E. faecalis*) (Diogo et al., 2017; Schuenck-Rodrigues et al., 2020). The MIC of nanoemulsion containing zinc phthalocyanines (ZnPc-NE) against *E. faecalis* was only 1.09 μg ml^−1^. Under a 60 or 90 s irradiation, Zn(II)chlorin e6 methyl ester (Zn(II)e6Me) could eliminate around 60% of the biofilm’s biomass. Sodium hypochlorite (NaClO) remains the most commonly used irrigant for root canal treatment worldwide. However, a higher concentration of NaClO with high antibacterial activity could cause severe complications. To address this issue, a visible light-guided root canal cleaning system consisting of a mixture of neutralized 0.5% NaClO solution and TiO_2_-x NPs was developed. The system produced various ROS including ^·^OH, ^·^Cl, and ^·^ClO, and after treatment with this system for 5 min, the killing rate of planktonic *E. faecalis* was 99.3% and that of biofilm was 100% (Liu et al., 2022). In another study, PTT utilizations with 1% NaClO solution showed superior performance in tooth root canal therapy (Duan et al., 2022). A novel D-A semiconducting conjugated polymer (PBDT-DIID) was designed, showing 70.6% photothermal conversion efficiency. The 1% NaClO solution with 25 μg ml^−1^ and 50 μg ml^−1^ could kill 99.7 and 99.6% of the bacteria, respectively. Meanwhile, the temperature elevation outside the root canal was controlled within 10°C, protecting periodontal membrane cells from damage.

### Periodontitis

Among all oral infectious diseases, researchers have studied the most about photodynamic materials for the treatment of periodontal disease. Different kinds of materials are designed according to the characteristics of the disease. Generally, the lesions formed in the periodontal pocket are irregular and periodontitis tends to occur in deep tissue. NPs with amphiphilic silane containing Chlorin e6 (Fe_3_O_4_-silane@Ce6/C6) were designed to kill periodontitis-related pathogens. The ratio metric fluorescence of Ce6/C6 could monitor the aPDT effect of Ce6 and Fe_3_O_4_ and realize the magnetically targeting function. Near-infrared (NIR) light has a strong penetration ability in tissue. Taking advantage of this feature, nanomaterials based on upconversion nanoparticles (UCNPs) were developed to combat periodontitis-related pathogens such as *P. gingivalis*, *Prevotella intermedia*, and *F. nucleatum*. NaYF_4_-Mn30%@Ce6@silane reached the maximum CFU reduction by more than 2 log (Zhang et al., 2019). Another work realized the conversion from NIR light to ultraviolet light and triggered the luminescence catalytic material titanium dioxide (TiO_2_), to realize the deep aPDT. UCNPs@TiO_2_ could reduce the three single-species biofilm CFU by about 4 orders of magnitude. Deep tissues are often in a hypoxic state, and most of the pathogenic bacteria of periodontitis are anaerobic bacteria. The process of PSs producing ^1^O_2_ *via* the type II mechanism needs consuming oxygen. Therefore, an oxygen self-sufficient nanoplatform (Fe_3_O_4_@Ce6-MnO_2_) was designed to solve the above problems (Sun X. et al., 2021). MnO_2_ nanolayer could react with hydrogen peroxide in the environment to generate O_2_ to enhance the efficacy of aPDT. In addition, since not all aPDT could achieve the ideal treatment effect, aPDT-based synergistic therapies were considered as strategies. Tinidazole-loaded TAT-Ce6 conjugate showed remarkable synergistic anti-periodontitis effects of aPDT and antibiotic therapy (Li et al., 2021a). Rapid killing of periodontal pathogens can also be achieved through the synergistic effect of aPDT and released ions. This 2D MOF CuTCPP-Fe_2_O_3_ nanosheet could also alleviate inflammation and promote angiogenesis (Li J. et al., 2021). However, too much ROS could cause serious oxidative stress and may aggravate tissue damage. The temporal sequence of ROS generation and scavenging is used to realize the antibacterial and anti-inflammatory functions. In the first stage, PSs produce ROS to kill bacteria. After that, the ROS scavenger removed excess ROS which is beneficial to reduce inflammation and tissue repair. Materials reported with the above functions are CeO_2_@Ce6 with 4 log CFU reduction and cyanobacteria loading Ce6 and Cu_5.4_O (CeCyan-Cu_5.4_O) with about 100% killing of the anaerobic bacteria biofilm (Sun Y. et al., 2021; Wang et al., 2022).

Hydrogels are the most popular polymers applied as delivery scaffolds for tissue generation and function as carriers for PTA loading. Au NPs such as Au nanorods (AuNRs), Au nanobipyramids (Au NBPs), and gold nanocages (GNC), as common PTAs, have high photothermal conversion efficiency. Chitosan hydrogels embedded with AuNRs showed significant anti-biofilm activity (Bermudez-Jimenez et al., 2020). With an increase of 10°C, there was a 5–8 log reduction against *Streptococcus oralis* (*S oralis*) and *E faecalis* biofilms. In another study, minocycline-loaded Au NBPs@SiO_2_ was incorporated into hydrogels and the synergetic antibiotic and photothermal treatment could kill 90 and 66.7% of *P. gingivalis* on the 3rd and 5th days (Lin et al., 2020). Zhang et al. (2020) designed a nano-antibiotic platform (TC-PCM@GNC-PND), which is composed of GNC, phase-change materials (PCM), poly(N-isopropylacrylamide-co-diethylaminoethyl methacrylate) (PND), and tetracycline (TC). Two thermos-sensitive interactions of PCM and PND could precisely control the release of the encapsulated drugs. Meanwhile, the platform could act as injectable hydrogel *in situ*, promoting the retention of antimicrobial agents in local infectious sites. In addition, chlorhexidine-incorporated hydrogels composed of curdlan and PDA were fabricated for periodontal treatment (Tong et al., 2020). The bactericidal effect comes from the synergistic effect of photothermal action and antibacterial agent. The best antibacterial rate is as high as 99.9%.

While considering the antibacterial treatment of periodontitis, attention should also be paid to inflammatory regulation. A baicalein-loaded mesoporous Prussian blue (MPB-BA), which has antioxidant, anti-inflammatory, and antibacterial effects, was designed for bacteria-induced periodontitis treatment (Tian et al., 2022). No CFU could be found on blood agar plates of the MPB-BA group, suggesting excellent antibacterial activity. Macrophages could be switched to M2 phenotype and MPB-BA-regulated inflammation *via* the inhibition of the Nrf2/NF-κB pathway. The multimodal synergistic treatment of aPDT/PTT combined with other treatments can further improve the bactericidal and anti-biofilm effects and can obtain multiple therapeutic effects at the same time. Self-assembled NPs (sPDMA@ICG) with aPDT/PTT and ion therapy were applied for the alveolar bone resorption inhibition and inflammatory alleviation of periodontitis (Shi et al., 2021). The star-shaped brush poly(2-(dimethylamino)ethyl methacrylate) (sPDMA) with a positive charge could make whole NPs tightly absorbed on the surface of *P. gingivalis*. NPs containing 10 μg ml^−1^ ICG almost inhibited the bacterial and biofilm growth due to aPDT/PTT effects. Another research is on aPDT/PTT and nitric oxide (NO) gas synergistic therapy platforms for biofilm eradication and inflammation regulation against periodontal diseases (Qi et al., 2022). AuNRs and ICG acted as heat-source, controlling the generation of NO. Meanwhile, ICG could produce ROS under NIR irradiation. The nanoplatform showed excellent antibiofilm functions with an about 4-log reduction in CFU of multi-species biofilm. In addition, expressions of adhesin molecule genes and virulence factor genes of *P. gingivalis* significantly downregulated after treatment.

In addition, LLLT or PBM is a therapy that utilizes low-level laser irradiation on cells or tissues to regenerate tissue, reduce inflammation, and reduce pain (He et al., 2021). One study showed that both aPDT and LLLT treatments improved the clinical parameters of periodontal disease and there were no significant differences between the two treatments (Freire et al., 2020).

### Other oral infectious diseases

Both aPDT and PTT were proven to be effective treatments for peri-implantitis (Wang et al., 2019; Xu B. et al., 2022), oral candidiasis (or *Candida albicans*; Dos Santos et al., 2019; Vila-Nova et al., 2022), oral lichen planus (Al-Maweri et al., 2018), jawbone infections (or methicillin-resistant *S. aureus*) (Araujo et al., 2018; Nie et al., 2022), and alveolar repair (Ervolino et al., 2019). However, research on these diseases mainly focused on killing pathogens *in vitro* or evaluating the evaluation of commercially available PSs for clinical treatment. The development of therapeutic materials for these diseases is required in the future.

## Present problems in current materials and future perspectives

Although research and development of aPDT and PTT materials have entered a climax in recent years, there are still some problems that have to be mentioned. For example, as far as PSs or PTAs are concerned, the concentration, light intensity, and irradiation time used are also different due to different materials. Notably, high-dose NIR light could activate Transcription Factor-4 (ATF-4)-mediated endoplasmic reticulum stress and autophagy, inducing NIR light phototoxicity (Khan et al., 2015). There is currently no unified standard or safety threshold for these parameters. Still, most experiments remain *in vitro* experiments, and there is still a long way to go before product transformation. Compared with the development of photonics-based materials in other medical disciplines, the development in the field of stomatology is still in its infancy. The oral microenvironment is extremely complex, such as different pH, temperature tolerance of different parts, fluidity of saliva, and gingival crevicular fluid. Therefore, combined with the characteristics of oral infectious diseases, personalized photonics-based oral materials and oral materials integrated with diagnosis and treatment will be the development trend and the focus of future research. It is expected that more products can be transformed into clinical practice for the benefit of human beings.

## Author contributions

SH: writing-original draft and data curation. MQ: conceptualization, methodology, visualization, supervision, and writing-reviewing and editing. YC: writing-original draft. All authors contributed to the article and approved the submitted version.

## Conflict of interest

The authors declare that the research was conducted in the absence of any commercial or financial relationships that could be construed as a potential conflict of interest.

## Publisher’s note

All claims expressed in this article are solely those of the authors and do not necessarily represent those of their affiliated organizations, or those of the publisher, the editors and the reviewers. Any product that may be evaluated in this article, or claim that may be made by its manufacturer, is not guaranteed or endorsed by the publisher.
